# EZpred: Improving Deep Learning‐Based Enzyme Function Prediction Using Unlabeled Sequence Homologs

**DOI:** 10.1002/advs.78703

**Published:** 2026-11-10

**Authors:** Chengxin Zhang, Quancheng Liu, Lydia Freddolino

**Affiliations:** ^1^ Gilbert S Omenn Department of Computational Medicine and Bioinformatics University of Michigan Ann Arbor Michigan USA; ^2^ Department of Biological Chemistry University of Michigan Ann Arbor Michigan USA

**Keywords:** deep learning, enzyme commission (EC) number, protein function prediction, sequence homologs

## Abstract

Features extracted from sequence homologs significantly enhance the accuracy of deep learning‐based protein structure prediction. Indeed, homolog‐aware models such as AlphaFold generally produce more accurate protein structures compared to single sequence‐based methods like ESMfold. In contrast, features from sequence homologs are seldom employed for deep learning‐based prediction of the biological functions of a protein, such as Gene Ontology terms or Enzyme Commission (EC) numbers. Although a small number of models also incorporate function labels from sequence homologs, they cannot utilize features extracted from homologs that lack function labels. To address this gap, we developed EZpred, which is the first deep learning model to use sequence homologs by extracting their sequence features while ignoring any function labels. Starting with the target sequence and homologs identified by MMseqs2, EZpred extracts sequence features using the ESM‐C protein language model. These features are then fed into a deep learning model to predict the EC numbers of the target protein. For test enzymes with < 30% sequence identity to any training proteins, the F1‐score of EZpred EC number prediction is at least 34% higher than state‐of‐the‐art EC number prediction models. These results demonstrate the strong positive impact of sequence homologs in deep learning‐based enzyme function prediction.

## Graphical abstract

Schematic of the EZpred pipeline, which provides high‐confidence predictions of enzyme function using a combination of sequence alignments, structural alignments, and deep learning. EZpred can be used to provide substantial increases in the richness of protein function annotations at genomic scales.

## 1 Introduction

In the past five years, the development of deep learning models for high accuracy protein structure prediction is the most important breakthrough in computational biology. Notable successful structure prediction models include AlphaFold [1, 2], RoseTTAFold [3], and D‐I‐TASSER [4]. In all these models, the target protein sequence is searched through one or more sequence databases to identify sequence homologs. Although these homologs do not have known structures, their alignment is still used to extract input features for the deep learning models. As the entry point of protein structure prediction, homologous sequence search and alignment have a strong impact on the accuracy of predicted protein structure models [5, 6]. On the other hand, single sequence‐based protein structure models such as ESMfold [7] that extracts features from the target sequence alone generally have worse accuracy than models that use homologous sequence alignments, as shown by recent Critical Assessment of Structure Prediction 15 (CASP15) challenge [8].

Although the usage of homologous sequences has been highly successful in deep learning‐based protein structure prediction, similar success has not been replicated in the prediction of protein functions, especially Gene Ontology (GO) terms and Enzyme Commission (EC) numbers. Traditionally, protein function prediction was achieved by transferring function annotations from homologous detected by sequence and structure searches. For example, GOtcha [9], Blast2GO [10], ConFunc [11], PFP [12], and CombFunc [13], and GoFDR [14] extracts GO terms from templates detected by sequence search performed using BLASTp [15], PSI‐BLAST [15] or DIAMOND [16]. Our recent study shows that when using optimized sequence search parameters, BLASTp, DIAMOND and MMseqs2 [17] can all achieve comparable performance [18]. Apart from sequence templates, COFACTOR [19, 20], StarFunc [21] and ProFunc [22] use function annotations from templates detected by structure alignment performed using TM‐align [23], Foldseek [24], SSM [25] and Jess [26]. Apart from sequence templates and structure templates, other template information such as protein‐protein interaction partners and protein domain families is also used for function prediction [27, 28, 29, 30, 31].

While traditional protein function annotation tools are mainly based on template search, most deep learning‐based methods for protein function prediction extracts features solely from the target protein sequence. To represent the target protein sequence, some models such as DeepGO [32], DeepGOplus [33] and ProteInfer [34] use one‐hot encoding, while other models such as ATGO+ [35], AnnoPRO [36], DeepGO‐SE [37] and CLEAN [38] uses protein language models such as ESM [7] or ProtT5 [39]. A small number of deep learning models such as SPROF‐GO [40] and GraphEC [41] also use function annotations from sequence homologs to improve prediction accuracy. Recently, sequence features from the multiple sequence alignments (MSAs) of unlabeled sequence homologs –i.e., sequences used without regard to annotations relevant to the particular prediction being made, whether present or absent – have been used to annotate the residue‐level functions, e.g., binding site residues [42], variant effects [43], and stability change upon single residue mutations [44]. To the best of our knowledge, though, similar MSA information has not been used for function prediction at the protein level, e.g., GO terms and EC numbers. When using sequence homologs for prediction of functions at the protein‐level, both traditional template‐based tools and deep learning methods only use sequence templates with known functions. This is different from deep learning‐based protein structure prediction, where sequence homologs without known structures play an important role.

To close this gap, we propose EZpred, the first deep learning model that improves protein‐level function predictions using sequence homologs without regard to their function labels (which may or may not be present). It is specifically designed to predict enzyme functions in the form of EC numbers. EZpred searches the target enzyme sequence through a sequence database by MMseqs2 to detect enzyme homologs. Sequence features of the target protein and enzyme homologs are derived from the last three layers of the ESM Cambrian (ESM C) [45] protein language model. These features are average‐pooled along the sequence and concatenated together. The concatenated features are fed into a multi‐layer perception (MLP) network to predict EC numbers. It should be noted that our deep learning model only uses homologous enzymes for sequence feature derivation during inference time, and ignores the EC number labels of these homologs. This model is therefore fundamentally different from earlier methods such as COFACTOR and GraphEC that aggregate functional labels from homologs. Meanwhile, the target protein sequence and structure are used to search template database using MMseqs2 and Foldseek. EC numbers derived from templates are then combined with deep learning‐based prediction to derive the final prediction (Figure 1); our ablation analysis demonstrates that the improved performance of EZpred arises due to a combination of the inclusion of unlabeled sequence homologs, the integration of both sequence‐based and structure‐based templates, and specific architectural decisions regarding the treatment of rare EC terms and integration of PLM data. Overall, we show that EZpred provides highly accurate predictions of enzymatic activities that can be used to guide future experiments, and at the same time, establish the boundary that it should not be used to predict mutational effects (a fundamentally different task).

**FIGURE 1:**
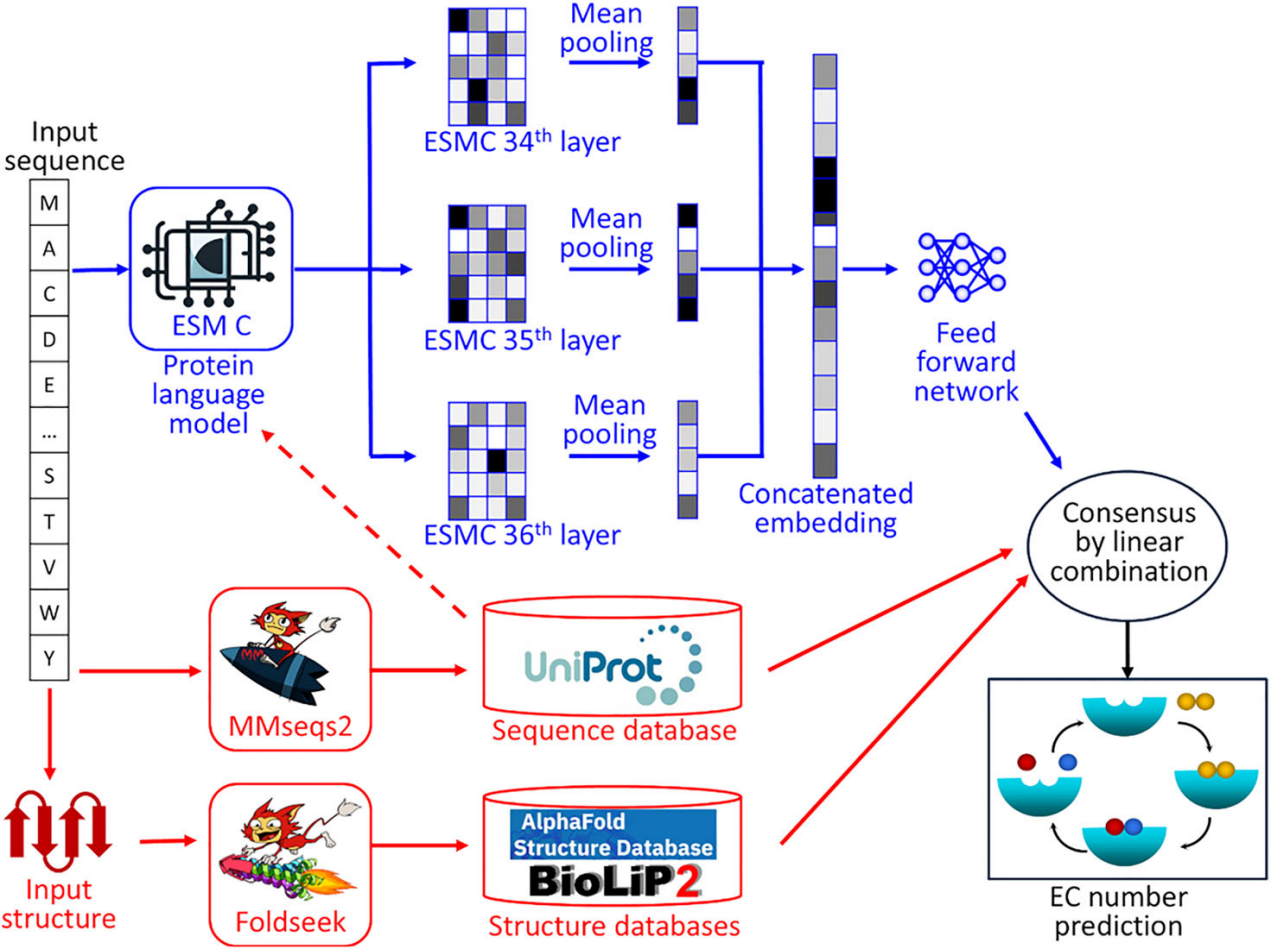
The EZpred pipeline. Blue indicates the deep learning (DL) part of EZpred, while red indicates the template‐based part of EZpred.

## 2 Results

### 2.1 Dataset

EZpred was trained on 30592 enzymes curated from UniProt release 2023_02. To collect enzymes, we extracted the EC number annotations based on the “DE”, “CC” and “DR BRENDA” fields of the plain text format UniProt data. Additionally, if the UniProt protein did not have EC number annotation but has Rhea [46] reaction annotation, we converted the Rhea ID to EC number using the rhea2ec mapping (https://ftp.expasy.org/databases/rhea/tsv/rhea2ec.tsv). Since these EC number annotations may come from either experiments or computational studies, we extracted the subset of experimental EC number annotations based on the Evidence and Conclusion Ontology (ECO) term associated with the EC number or Rhea ID annotation, where ECO:0000305 and ECO:0000269 indicate experimental annotations. Additionally, if the UniProt protein had GO annotations that can be converted to EC number using the ec2go mapping (http://release.geneontology.org/2024‐04‐24/ontology/external2go/ec2go), and the GO term is annotated with an experimental evidence code (EXP, IDA, IPI, IMP, IGI, IEP, HTP, HDA, HMP, HGI, HEP, TAS, or IC), we also collected this experimental annotation. These training proteins were clustered by CD‐HIT [47] using 60% sequence identity cutoff. We then partitioned the sequence clusters into 5 folds, stratified by EC labels. Hyperparameters of EZpred were optimized by 5‐fold cross validation. Here, the CD‐HIT clustering was applied to ensure that highly similar training proteins will not be partitioned into different folds of training set in cross‐validation.

Based on the same UniProt release, we also collected non‐enzymes as the negative training set. To generate this dataset, we collected all UniProt proteins with at least one Molecular Function (MF) GO term annotated with an experimental evidence code. The following GO terms were excluded as they are too general: GO:0046872 “metal ion binding” as well as its child terms, GO:0005524 “ATP binding”, GO:0042802 “identical protein binding”, GO:0005515 “protein binding”, GO:0051260 “protein homooligomerization”, GO:0003676 “nucleic acid binding”. For the remaining proteins with at least one specific MF GO term annotation, we excluded enzymes that were annotated with EC numbers or any child terms of GO terms GO:0003824 “catalytic activity” or GO:0022857 “transmembrane transporter activity”. This resulted in 23,354 non‐enzyme training proteins. These negative training proteins were clustered by CD‐HIT using 60% sequence identity cutoff. We also then partitioned sequence clusters into 5 folds, stratified by EC labels, to perform 5‐fold cross‐validation.

To generate an independent test set, we collected 753 enzymes with new EC number annotation in UniProt release 2024_02 but no EC number, Rhea ID or MF GO terms that can be mapped to EC numbers as of UniProt release 2023_02. Among these enzymes, 33 of them are present in UniProt 2023_02 under a different UniProt accession, but identical gene name, organism and sequence. After excluding these duplications, the final test set consists of 720 enzymes. This test is much larger than the Price‐149 and New‐392 test sets (with 149 and 392 enzymes, respectively) originally used to develop the ProteInfer [34] and CLEAN [38] models, as well as the CAFA3 preliminary evaluation test set [48], which includes 649 proteins with MF GO terms. The test spans across all major enzyme classes and taxonomy divisions and has a similar sequence length distribution as the training set (Figure S1).

We also prepared a negative test set with 625 proteins that have new experimental MF GO term annotations to confirm non‐enzyme activity in UniProt release 2024_02 but without such GO terms in UniProt release 2023_02.

Similar to the CAFA challenge [48], the performance of EC number prediction is mainly evaluated by the protein‐centric F1‐score, which is the harmonic average of precision and recall; a higher F1‐score indicates more accurate prediction. For protein *t*, we define the precision and recall:

$$\;Precision_{t}=\frac{\sum_{q=1}^{Q}y_{true}\left(t,q\right)\cdot y_{pred}\left(t,q\right)}{\varepsilon +\sum_{q=1}^{Q}y_{pred}\left(t,q\right)}$$

$$\;Recall_{t}=\frac{\sum_{q=1}^{Q}y_{true}\left(t,q\right)\cdot y_{pred}\left(t,q\right)}{\varepsilon +\sum_{q=1}^{Q}y_{true}\left(t,q\right)}$$

here, ε = 1E‐16 is a small pseudo‐count to avoid division by zero; *y_true_* (*t*,*q*) indicates whether protein *t* has EC number *q*; and *y_pred_* (*t*,*q*) is the predicted probability of EC number *q* for protein *t*. *Q* is the total number of labels, i.e., EC numbers. For all *T* test proteins, overall F1‐score can be defined as:

$$F1=\frac{2}{T/\sum_{t=1}^{T}Precision_{t}+T/\sum_{t=1}^{T}Recall_{t}}$$

### 2.2 Overall Performance

The performance of EZpred and the pretrained models of 8 existing machine learning‐based EC number prediction methods on 720 enzymes are evaluated by the F1‐scores of predicting the first digit, the first two digits, the first three digits and all four digits of the EC number annotation. Among the existing EC number predictors, ProteInfer [34], ECPICK [49] and ECPred [50], DeepEC [51] extract features from the raw protein sequence; CLEAN [38], and DeepECtransformer [52] use protein language models [39, 53] to extract features from the protein sequence. While these 6z methods only use the protein sequence for feature extraction, GraphEC [41] and CLEAN‐Contact [54] use both the 3D structure (represented by a contact map) and the protein sequence (featurized by protein language model) to extract the features.

Overall, all benchmarked methods can relatively accurately predict the first three digits of EC numbers with F1‐score ≥0.506, where EZpred achieves the highest accuracy (F1‐score = 0.887, Figure 2). On the other hand, predicting all four digits EC number is much more challenging, where the F1‐score ranging from 0.786 (EZpred) to 0.425 (DeepEC). The F1‐score of EZpred is 27% and 31% higher than CLEAN and GraphEC (Wilcoxon test p‐values 3.51E‐20 and 2.89E‐31, respectively, Table S1), which are the two most accurate methods. Evaluation by Area Under the Precision‐Recall Curve (AUPRC) reaches similar conclusion (Figure S2). This performance difference is unlikely to be caused by the difference in EC number definitions used by different methods. For example, 93% of training labels (i.e., EC numbers that can be predicted by different models) are shared between EZpred and CLEAN. In fact, even if we switch the EC number definitions from that of EZpred to that of CLEAN, EZpred still outperforms all existing methods in this benchmark test (Figure S3). The advantage of EZpred is not caused simply by training the model with a more recent dataset compared to other methods. For example, when we retrained the CLEAN model using the same training set as we did for EZpred, the deep learning model of EZpred and the retrained CLEAN model achieve F1‐scores of 0.792 and 0.607, respectively, when evaluating on all four digits of EC number prediction on the test EZpred test set.

**FIGURE 2:**
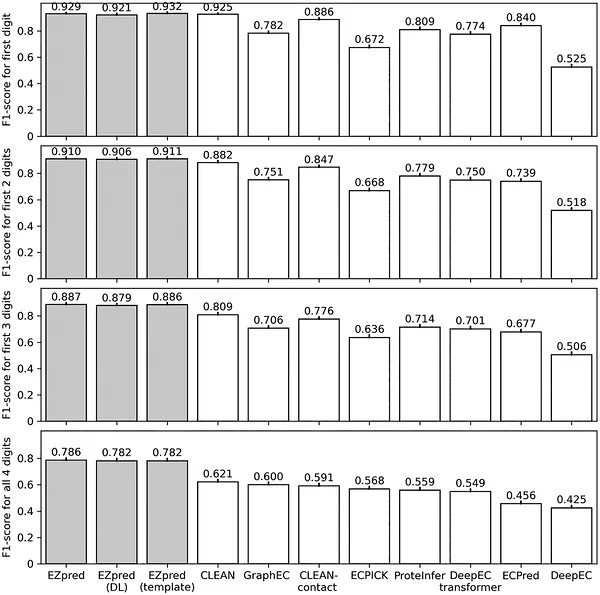
F1‐score of EC number prediction for 720 test enzymes. EZpred (template) and EZpred (DL) indicates the template‐ and deep learning‐based components of EZpred. Bold font highlights the highest F1‐score. The error bars indicate the standard error of mean (SEM) of per‐protein F1‐score values.

This performance advantage generalizes well to different classes of enzymes, as EZpred is more accurate than all existing methods to predict 6 out of all 7 major enzyme classes in this benchmark (Table S1). EZpred is also able to predict rare enzymes, albeit with lower accuracy: for 4‐digit EC numbers that are associated with no more than four training proteins, EZpred achieves an F1‐score of 0.402, which is 15% and 136% higher than those of CLEAN and GraphEC, respectively (Figure S4). While the template‐ and deep learning‐based components of EZpred achieve a similar performance (F1‐score = 0.782), combining these two components results in an improvement of 0.5%. This improvement is modest, probably because both components have already achieved high performance, making further improvement difficult. Due to the usage of the fast structure search tool Foldseek, the structure template search component only takes approximately 1 min for the full test set, accounting for 56% of the total running time of EZpred (Table S2).

To further investigate the impact of sequence similarity on model performance, we partition the test set into “easy”, “medium”, and “hard” target sets, where their maximum sequence identity to the most similar training enzyme is in the range of 0.6∼1, 0.3∼0.6 and 0∼0.3, respectively (Figure 3). The performance of all tested methods is worse for targets with lower sequence similarity. For all three sets of targets, EZpred as well as its component methods outperform existing methods. When predicting all 4 digits of EC number, although the deep learning‐based component of EZpred slightly outperforms the template‐based component for easy and medium targets, the former is worse than the latter for hard targets (F1‐score = 0.598 and 0.638, respectively). This is probably because the deep learning‐based component only uses sequence information while the template‐based component also uses the tertiary structure, which allows more sensitive function detection by the latter component for challenging targets.

**FIGURE 3:**
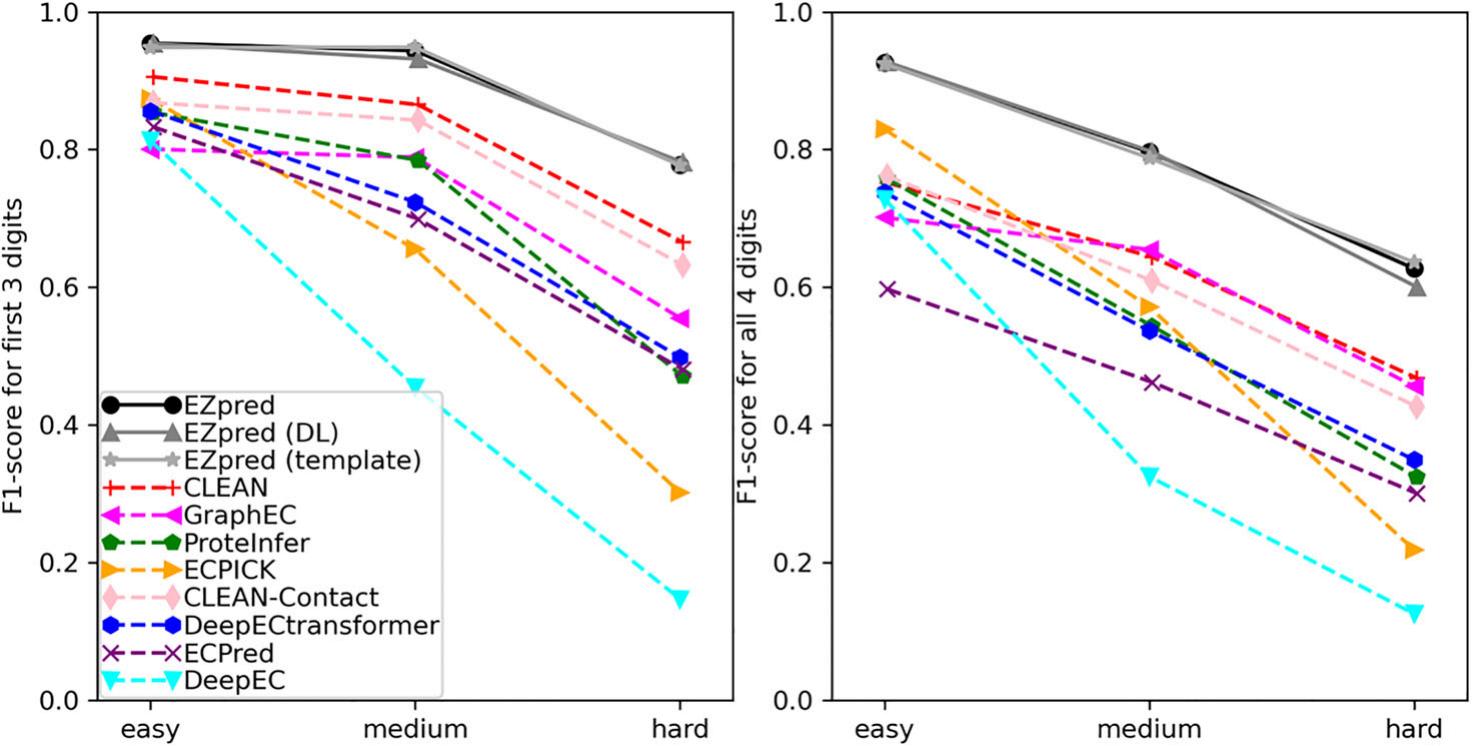
Performance of EZpred and existing methods for predicting the first 3 and all 4 digits of EC numbers on 219 “easy” targets, 248 “medium” targets and 253 “hard” targets among the 720 testing enzymes. The “easy”, “medium”, and “hard” test subsets correspond to the maximum sequence identity between the test enzyme and the most similar training enzyme in the range of 0.6∼1, 0.3∼0.6 and 0∼0.3, respectively. Here, the sequence identity is defined as the number of identical residues in the Needle‐Wunsch global sequence alignment (https://zhanggroup.org/NW‐align/) divided by the maximum sequence length between the training and testing enzyme. We did not impose a minimum alignment length of coverage threshold when performing the alignment.

We investigated whether the strong performance of the EZpred deep learning model on hard targets reflects transfer of function from structurally related training proteins or generalization of the model to non‐homologous proteins. To this end, we performed a more stringent test on a subset of very hard targets with a TM‐score≤0.6 to any training proteins (reflecting the absence of any similar structures in the test set). Benchmarking on this stringent subset confirms that the deep learning component of EZpred generalizes well to non‐homologous test cases and consistently outperforms all existing methods, achieving an F1‐score >17% higher (Table S3).

### 2.3 Enzyme vs Non‐Enzyme Prediction

In many real case scenarios, we do not know whether the input protein is an enzyme or non‐enzyme. Therefore, a practical enzyme function predictor should be able to differentiate enzymes versus non‐enzymes. The deep learning‐based component of EZpred has a separate label (EC number 0.‐.‐.‐) for non‐enzymes. DeepECtransformer and ECPred also predict similar non‐enzyme label (“None” for DeepECtransformer and “non Enzyme” for ECPred). We compare the performance of EZpred, DeepECtransformer and ECPred in differentiating enzymes from non‐enzymes. On the full testing dataset containing 720 enzymes and 625 non‐enzymes, the F1‐scores for predicting whether a target protein is an enzyme is 0.942, 0.753 and 0.909 for EZpred, DeepECtransformer and ECPred, respectively (Table S4). This shows EZpred is much more sensitive in identifying enzymes than existing methods.

We wondered whether the apparent higher accuracy of enzyme versus non‐enzyme prediction by EZpred is due to difference of definitions for non‐enzymes by different methods. In EZpred, non‐enzymes are defined as proteins with known molecular function GO terms and no known catalytic activities. On the other hand, in DeepECtransformer [52] and ECPred [50], non‐enzymes are defined as proteins with high UniProt annotation scores and no known catalytic activities, regardless of whether their molecular function is known or not. Therefore, we generated another negative dataset with 377 non‐enzyme proteins using the same procedure as ECpred. Specifically, we include new UniProt proteins that are present in release 2024_02 but absent in release 2023_02 with annotation scores of 4 and 5 and no EC numbers. When using this new dataset, the F1‐scores for predicting whether a target protein is an enzyme are 0.913, 0.790 and 0.899 for EZpred, DeepECtransformer and ECPred, respectively. These data show that the high accuracy of enzyme/non‐enzyme classification is not a result of different definitions of non‐enzymes, but rather, that EZpred achieves higher performance using either definition.

To train EZpred for enzyme/non‐enzyme classification, the deep learning component is trained to predict the 5586 EC numbers and the non‐enzyme label. This set up result in higher enzyme/non‐enzyme classification accuracy than EZpred trained as a binary classifier for enzyme versus non‐enzyme (Table S4). This is because training on the 5586 EC numbers labels serve as auxiliary loss that positively contribute to the enzyme/non‐enzyme classification task.

### 2.4 Ablation Study

EZpred is different from many other deep learning‐based protein function prediction models in the choices of different hyperparameters and feature representations. To quantify the contributions of these choices, we perform a set of controlled experiments.

First, many methods exclude rare function labels during training. For example, InterLabelGO [55] excludes GO terms with less than 10 training proteins; DeepECtransformer excludes EC numbers with less than 100 training enzymes. On the other hand, EZpred includes any EC number in the training set, even if there is only one training protein associated with the EC number. Including all EC numbers results in 5% and 16% higher F1‐score than the same model architecture including EC numbers with at least 5 or 10 training enzymes (Table 1). In this ablation study, the improvement from including rare EC number labels is not caused by a larger training set. In fact, the training set is the same no matter whether rare labels are included or not. For example, the EC number 1.1.1.374 “UDP‐N‐acetylglucosamine 3‐dehydrogenase” is associated with only one training enzyme (UniProt accession Q6M0B9). When the training set excludes rare labels, the training enzyme Q6M0B9 is still included for the EC numbers 1.‐.‐.‐ “Oxidoreductases”, 1.1.‐.‐ “Acting on the CH‐OH group of donors” and 1.1.1.‐ “With NAD(+) or NADP(+) as acceptor”, while the rare label 1.1.1.374 is excluded. A probable reason that including rare function labels improves EZpred but not previous methods is our usage of a ranking‐based loss function (See Methods). This ranking‐based loss makes the learning of sparse labels effective, which may not be true for previous methods that use binary cross entropy‐based loss.

**TABLE 1:** F1‐score of predicting all 4 digits of EC number prediction by different versions of deep learning component of EZpred on all 720 test enzymes, including 219 “easy” targets, 248 “medium” targets and 253 “hard” targets.

EZpred (DL) versions	F1‐score			
	All	Easy	Medium	Hard
Default (include all EC numbers; search enzyme‐only database for homologs; use last three layers of ESM C)	**0.782**	0.927	**0.797**	**0.600**
Only include EC numbers with ≥5 training proteins	0.737	0.867	0.745	0.597
Only include EC numbers with ≥10 training proteins	0.675	0.817	0.662	0.543
Search enzyme and non‐enzyme databases for homologs	0.770	0.923	0.782	0.589
Do not search sequence database for homologs	0.756	0.904	0.765	0.578
Use last one ESM C layer	0.763	**0.928**	0.792	0.567
Use last two ESM C layers	0.772	**0.928**	0.788	0.587
Use last four ESM C layers	0.768	0.924	0.778	0.596
Use last ten ESM C layers	0.767	0.920	0.789	0.581
Use last three ESM‐1b layers	0.754	0.923	0.770	0.564
Use last three ProtT5 layers	0.773	0.922	0.791	0.598

Second, existing deep learning models usually extract sequence features from the target protein sequence itself. On the other hand, EZpred extracts sequence features from both the target protein and sequence homologs detected by MMseqs2 [17] sequence search. Including sequence homologs results in 3% improvement in the F1‐score for EC number prediction. This improvement caused by inclusion of homologous sequence features is also observed for another protein language model, ESM2 [7], which was used in our previous work [55] (Figure S5). Additionally, including only enzymes in the sequence database for MMseqs2 sequence search results in 1% greater F1‐score than including both enzymes and non‐enzymes in the database (Table 1). It is not surprising that using a mixture of enzymatic and non‐enzymatic homologs does not result in more effective feature extraction. EZpred performs two related tasks: first, EZpred predicts whether a protein is an enzyme; second, assuming that a protein is an enzyme, EZpred predicts its EC number. For the first task, the inclusion of enzyme‐only homologs provides a reasonable prior for the method to differentiate enzymes versus non‐enzymes simply by existences of homologs. For the second task, inclusion of non‐enzymes introduces more noise as they are not associated with EC numbers.

Third, with very few exceptions [35, 55], most existing models that use protein language models for sequence feature extraction only use the output from the last layer. On the other hand, when EZpred uses the ESM C model for feature extraction, outputs from the last three layers of ESM C are all used. The ablation study shows that using these three layers results in 2% greater F1‐score than using only the last layer (Table 1). Using two or more than four ESM C layers also leads to worse performance. These data are consistent with our previous work on protein GO term prediction [55].

Fourth, using the ESM C protein language model, results in 1% and 3% higher F1‐scores than using ProtT5 [39] and ESM‐1b (Table 1). These show that the choice of protein language model for feature generation is important for the performance of function predictor.

Fifth, since EZpred performed 5‐fold cross validation to tune hyper‐parameters during training, 5 models are trained for EC number prediction. During inference, the prediction results of these models are averaged to get the final prediction. These five models can be considered replicated experiments, with similar performance on the test set (Table S5). The final prediction ensembling the five models achieved ≥3% higher F1‐score than any individual model.

Sixth, the template‐based component of EZpred combines both sequence search and structure search results from MMseqs2 and Foldseek [24], respectively, where the structures of the target and templates proteins are from the AlphaFold protein structure database (AFDB) [56]. The combined template‐based component achieves 2% higher F1‐score than those using sequence and structure hits only, indicating that both sequence and structure templates are helpful for function prediction. We note that the combination of deep learning and template‐based components of EZpred only improves 4‐digit EC number prediction by 0.5% in F1‐score compared to the individual components. This improvement is small, because the two components are independently robust: since both components are far more accurate than baseline methods such as CLEAN, further improvement of either component is difficult due to a ‘ceiling’ effect. Nonetheless, the two components are still complementary. For example, the template‐based component outperforms the deep learning component for targets with modest structural similarity (0.2 < TM‐score ≤ 0.6 to training proteins) as the former can ensemble gather functional labels from a large set of low homology templates by structural retrieval. Yet, for very hard targets with TM‐score < 0.2, deep learning component outperforms template‐based components as the latter cannot identify useful templates (Figure S6).

### 2.5 A Case Study on Multi‐function Enzyme Annotation

As a case study, we investigated the EC number prediction for 7,8‐linoleate diol synthase from the fungus *Pyricularia oryzae* (UniProt accession: G4N4J5). This synthase is a bifunctional enzyme with two EC numbers: EC 1.13.11.60 “linoleate 8R‐lipoxygenase” and EC 5.4.4.6 “9,12‐octadecadienoate 8‐hydroperoxide 8S‐isomerase”. EC 1.13.11.60 converts linoleic acid (18:2n‐6) into 8‐hydroperoxy‐8(E),12(Z)‐octadecadienoic acid (8‐HPODE, Figure 4A), while EC 5.4.4.6 catalyzes the isomerization of 8‐HPODE to 7,8‐dihydroxy‐9(Z),12(Z)‐octadecadienoic acid (7,8‐DiHODE, Figure 4B). Similar to almost all existing EC number predictors, EZpred formulates the EC number prediction problem as a multi‐label classification problem, where each data instance (protein) can have one or more labels (EC numbers). Therefore, in theory, all 8 existing predictors should work for bifunctional enzymes. In practice, however, among the 8 existing predictors, none of them can predict EC 5.4.4.6 for this protein; only two predictors (ProteInfer and DeepEC) can correctly predict EC 1.13.11.60 (Figure 4C). On the other hand, both the template‐based and deep learning components of EZpred correctly predict EC 1.13.11.60, and the latter also correctly predicts EC 5.4.4.6. Meanwhile, both components make a false positive prediction EC 5.4.4.5 which catalyzes isomerization of 8‐HPODE to 5,8‐dihydroxyoctadeca‐9,12‐dienoate (5,8‐HiHODE) instead of 7,8‐DiHODE (Figure 4D). This is due to several Psi‐producing oxygenase A enzymes in the training set that performs EC 1.13.11.60 and 5.4.4.5 and have high similarity to the target enzyme (UniProt accessions: G5EB19, Q4WPX2, Q6RET3, Figure 4E). These three template enzymes are ranked third, fourth and fifth among all templates, leading to the partially incorrect prediction. The false positive prediction of EC 5.4.4.5 illustrates the specific challenge faced by the deep learning component of EZpred in detecting and utilizing functionally relevant templates. Moreover, our data show that combining the template‐based (F1‐score = 0.667) and deep learning (F1‐score = 0.500) components can lead to more accurate predictions (F1‐score = 0.800, Figure 4C).

**FIGURE 4:**
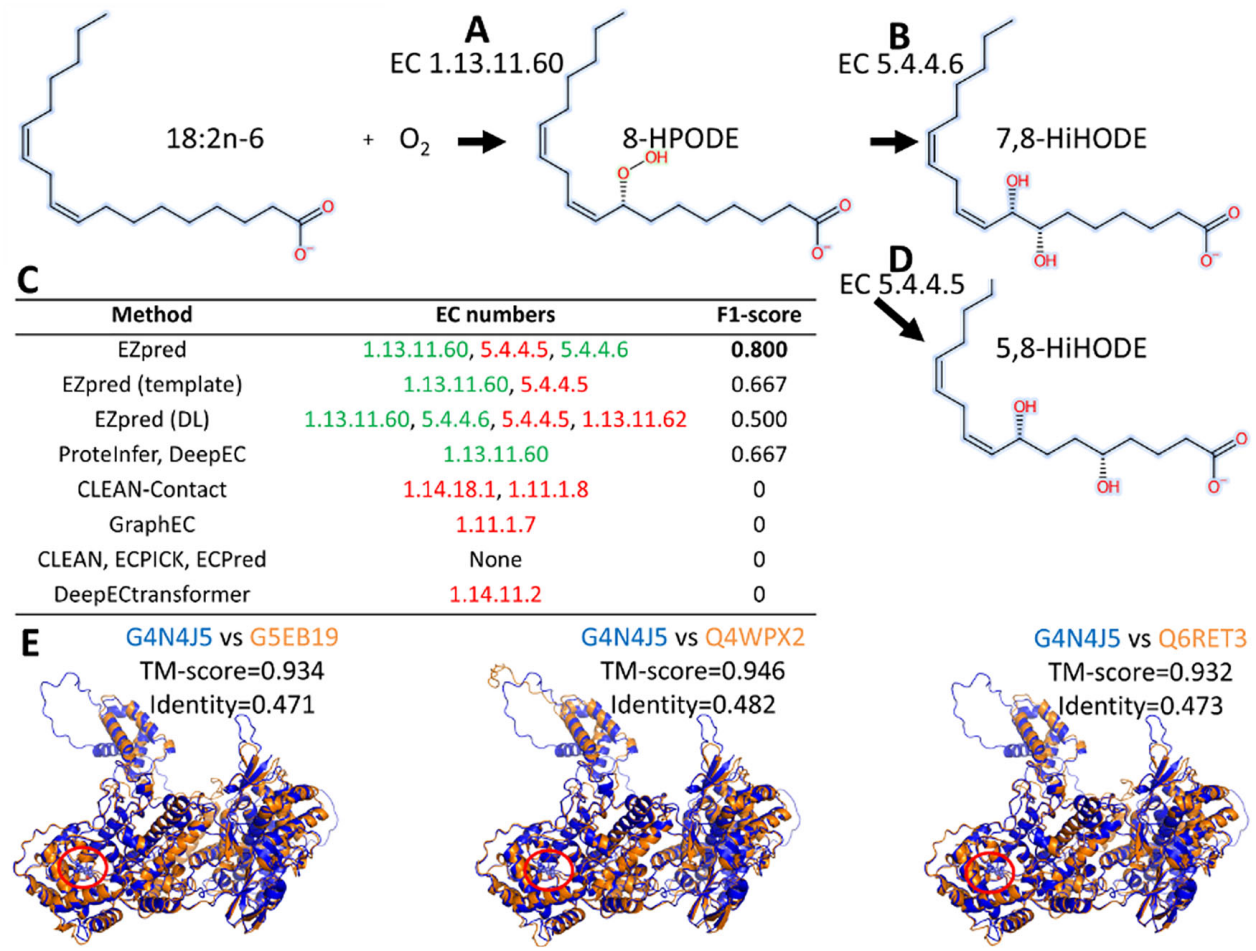
Case study of 7,8‐linoleate diol synthase (UniProt accession: G4N4J5). A. EC 1.13.11.60 “linoleate 8R‐lipoxygenase”. B. EC 5.4.4.6 “9,12‐octadecadienoate 8‐hydroperoxide 8S‐isomerase”. C. EC prediction by EZpred and existing methods. Green and red indicate correct and incorrect predictions. D. EC 5.4.4.5 “9,12‐octadecadienoate 8‐hydroperoxide 8S‐isomerase”. E. Structure superimposition between G4N4J5 (blue) and three structure templates (orange). Red circle indicates the catalytic active site.

### 2.6 A Case Study on Annotation of Biosynthetic Gene Clusters

The biosynthetic gene clusters (BGCs) of *Streptomyces* bacteria hold tremendous biosynthetic potential, and mining the biological products from this genus has given rise to the majority of natural product biomolecules with commercial or pharmaceutical applications [57]. However, a substantial challenge in the ongoing mining of streptomycetes and related bacteria for new natural products is that in many cases, the mapping between a particular BGC and its product cannot be definitely identified, despite the presence of specialized tools such as antiSMASH that merge a vast range of data to predict several key aspects of the regulation and likely products of BGCs [58]. Knowing the enzymatic functionalities encoded in a particular BGC is obviously essential to predicting likely products, yet in many cases the link between particular biosynthetic enzymes and the functions they enable remains a topic of active investigation even when the overall product of the BGC is known (e.g., [59]). Better predictions of the enzymatic functionalities encoded by each protein in a BGC would substantially assist both in de‐orphanizing the products of natural but cryptic BGCs, and facilitate re‐engineering of those BGCs for industrial applications and even novel product formation [60].

To provide an example of how EZpred can provide higher quality function predictions for BGC‐encoded enzymes giving rise to potentially useful natural products, we applied antiSMASH2 to the genome of *Streptomyces venezuelae* NRRL B‐65442, and examined the results for BGCs without a clear product (Figure 5). We selected as an example the region containing genes vnz_28150 – vnz_28265, which is predicted to encode a BGC producing some deazapyrimidine derivative, but for which no detailed product prediction is available and where many of the likely enzymes lack deep annotations. As seen in the schematic of this BGC in Figure 5A, EZpred provides high‐confidence predictions for the functions of several biosynthetic enzymes in this region, including many which are not even flagged as potential biosynthetic enzymes by antiSMASH (details in Figure 5B). EZpred provides substantially higher‐information function predictions than other existing resources for several enzymes in this cluster; for example, vnz_28180 is annotated by antiSMASH as a group I glycosyltransferase, and in UniProt as a galactosyltransferase, whereas EZpred provides a high confidence (0.79) prediction of EC 2.4.1.283 (2‐deoxystreptamine N‐acetyl‐D‐glucosaminyltransferase; see Figure 5C). This prediction not only provides substantially more insight into the likely role played by vnz_28180 itself, but actually recasts the most likely interpretation of the entire BGC. As 2‐deoxystreptamine N‐acetyl‐D‐glucosaminyltransferase enzymes are core components of aminoglycoside biosynthesis [61], our predictions (combined with the prior information from antiSMASH and the obvious presence of key enzymes like QueC/QueE [62]) suggest that the BGC under consideration would give rise to a compound such as huimycin or dapiramicin A which contain both 7‐deazaguanine and aminoglycoside moieties. The usage case here provides an illustrative example of how improved enzyme annotations can directly guide the search for new antibiotics and aid in de‐orphanizing natural biosynthetic pathways. While it is important to note that the information provided here (both regarding the functions of individual proteins and likely pathway end products) only constitutes a prediction, and must be experimentally validated, this application provides an example of how EZpred can guide experimental characterization of poorly annotated enzymes and even poorly annotated gene clusters.

**FIGURE 5:**
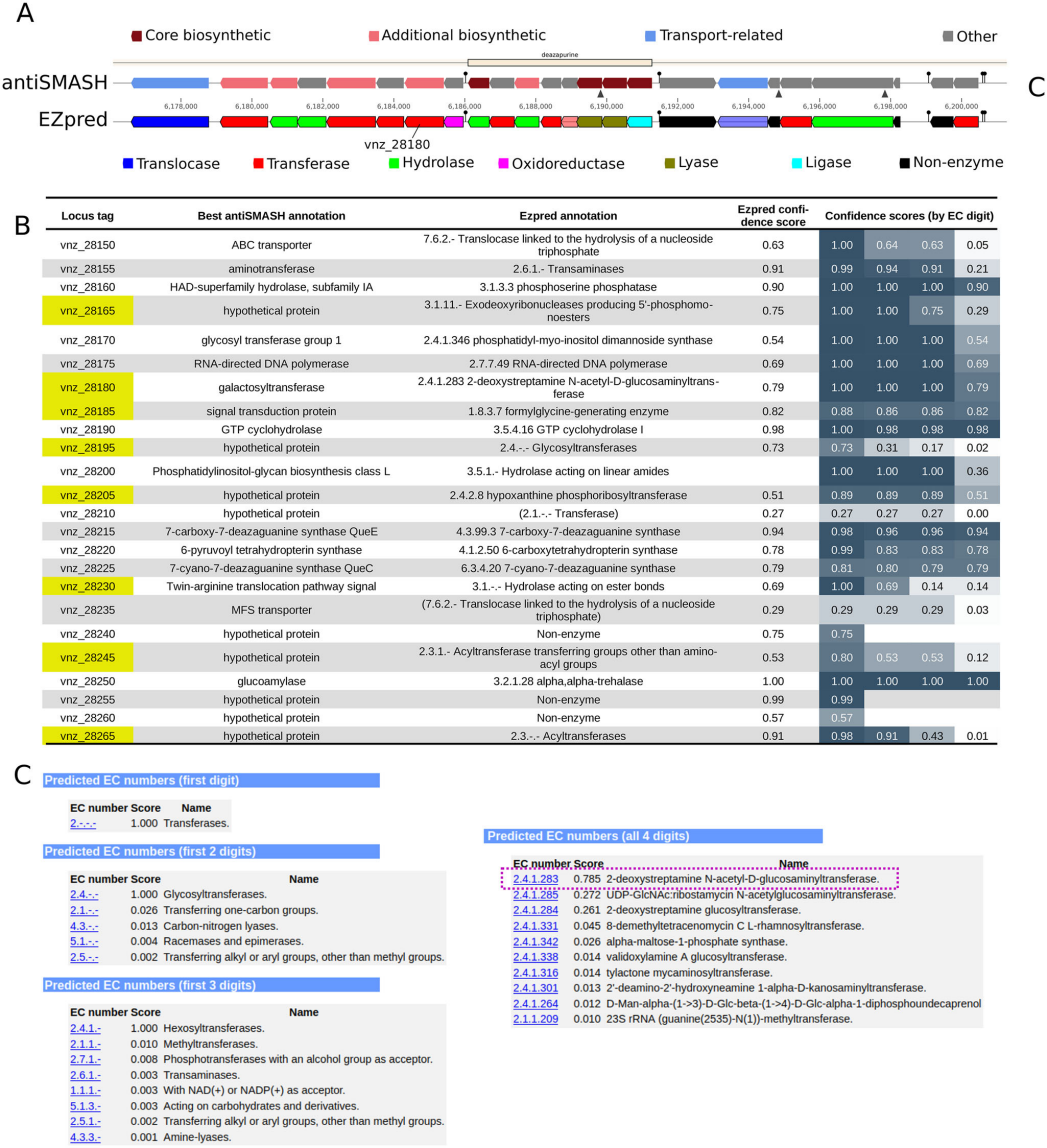
Case study of the annotation of a cryptic BGC from *Streptomyces venezuaelae* using EZpred. (A) Map of the BGC of interest, showing an overview of annotation categories from antiSMASH (top) vs. first‐digit EC terms from EZpred. (B) Detailed annotations from antiSMASH vs the most specific annotation for each gene with a confidence score above 0.5 from EZpred; the last four columns show the confidence scores for the strongest prediction at each EC digit level. (C) Full EC term predictions from EZpred for vnz_28180, with the most confident 4‐digit prediction (EC 2.4.1.283) shown in a purple box.

### 2.7 (Lack of) Impact of Mutations on Enzyme Function Prediction

Since the function of an enzyme is often determined mainly by a small number of ligand binding and active site residues, we wondered whether variations in critical residues of the input sequence would alter EZpred prediction. To this end, we compare the prediction scores for true positive EC numbers using wild type (WT) enzymes and corresponding mutants with the known catalytic active sites mutated to glycine (Figure S7). The deep learning predicted scores from EZpred for mutants are only slightly lower than those of WT (average prediction scores 0.855 and 0.857, respectively). This phenomenon is largely due to the mean pooling operation, which blurs residue level information of the input sequence, resulting in EZpred (as well as almost all existing deep learning models for similar annotation tasks) being sensitive to global sequence patterns but insensitive to local variations in the input sequence. Thus, these models are suitable for EC term predictions of most natural proteins, but not suitable for the task of distinguishing between functional and nonfunctional alleles of a particular protein.

This lack of impact of mutations on enzyme function prediction is not unique to EZpred. As a more concrete example, we studied the human aldehyde dehydrogenase ALDH2 (UniProt accession: P05091). ALDH2 is a mitochondrial enzyme that clears formaldehyde (EC 1.2.1.3 “aldehyde dehydrogenase (NAD(+))”). According to UniProt, single nucleotide polymorphisms (SNPs) on ALDH2 include 7 benign mutations (I41L, I41V, P92A, P92T, R94H, T244M, V304M) and one pathogenic mutation (E504K). Here, by drastically reducing enzymatic activity, E504K is associated with the aplastic anemia, mental delay, and dwarfism syndrome (AMEDS) [63, 64]. Yet, there is little difference in prediction score for its enzymatic function EC 1.2.1.3 using the wild type, benign mutations and pathogenic mutations (Figure S8). The same phenomena are also observed for CLEAN and GraphEC, the latter of which explicitly up‐weights residues important for catalytic activities when performing function inference. This case study highlights the general point that protein function prediction is a fundamentally different task from predicting the potentially pathogenic effects of point mutations; EZpred is designed for the former task, and the mean‐pooling that it employs largely removes information needed for the latter.

## 3 Discussion and Conclusion

In this work, we develop EZpred, a composite pipeline that combines deep learning, sequence homolog search and structure template alignment for the prediction of enzyme function. In a large‐scale benchmark dataset, EZpred consistently outperforms existing deep learning methods by >27% higher F1‐score when predicting the full 4‐digit EC number and >17% higher F1‐score when predicting whether a protein is an enzyme. Detailed analysis shows that including rare EC numbers, extracting features from sequence homologs, using the last three layers of the ESM C protein language model, and combining sequence and structure templates all contribute positively to the overall performance of EZpred.

Despite the high performance of EZpred, there are still two points that are worthy of further development efforts. First, while its template‐based component uses both sequence and structure for template search, EZpred does not use tertiary structure in its deep learning component. Integration of structural features [65, 66] may further improve the deep learning‐based component. Second, enzyme function is mainly determined by the active site residues and residues within the substrate/product binding pocket. Yet, the mean pooling procedure in the deep learning‐based component treats all residues equally. Therefore, future versions of EZpred will use higher weights for predicted active/binding site residues when pooling features for the deep learning component.

## 4 Materials and Methods

EZpred contains two components: the template‐based component and the deep learning‐based component (Figure 1).

### 4.1 Template‐Based Component of EZpred

The template‐based component of EZpred (red component in Figure 1) includes two parts: sequence template search and structure template search.

In the sequence template search step, the input protein sequence is searched by MMseqs2 through all training enzyme protein sequence using the following search parameters: “easy‐search –max‐seqs 4 –format‐output query, qlen, target, tlen, evalue, bits, alnlen, fident, qaln, qstart, taln, tstart ‐e 1e‐2”. For any EC number *q*, its sequence template‐based prediction score is calculated as:

$$\begin{array}{ccc} Cscore_{seq}\;\left(q\right) & = & w_{seq}\;\cdot \frac{\sum_{k=1}^{K\left(q\right)}ID_{k}\left(q\right)\cdot bitscore_{k}\left(q\right)}{\sum_{k=1}^{K}ID_{k}\cdot bitscore_{k}}+\left(1-w_{seq}\right)\\ & & \cdot \;\frac{\sum_{k=1}^{K_{site}\left(q\right)}ID_{k}\left(q\right)\cdot bitscore_{k}\left(q\right)\cdot SiteAligned_{k}\left(q\right)}{\sum_{k=1}^{K_{site}}ID_{k}\cdot bitscore_{k}\cdot SiteAligned_{k}} \end{array}$$

Here, *bitscore_k_* is the bit‐score for aligning the target protein to the *k*‐th template sequence. *ID_k_* is the global sequence identity for the *k*‐th template and equals the number of identical amino acid residues divided by the maximum between the target sequence length and template sequence length. *K* and *K_site_* are the total number of templates and the subset of templates with known active sites or substrate binding sites according to the UniProt database. In UniProt, active sites are annotated by UniProt through the “FT ACT_SITE” tag. Substrate binding sites are also detected from the UniProt data file by the following procedure: ChEBI IDs of the substrates and products of the catalytic activity is extracted from the “CC ‐!‐ CATALYTIC ACTIVITY” records; second, ligand binding residues are detected from the “FT BINDING” and “FT REGION” records; finally, the subset of binding residues associated with ChEBI of the substrate/product molecules via the ‘FT /ligand_id = “ChEBI:CHEBI:”)’ records are confirmed to be substrate binding residues. We then defined the catalytic site pocket by including all active site residues, substrate binding site residues, and all other residues whose sequence separations to the above residues are ≤ 2. *SiteAligned_k_* is the portion of catalytic site pocket residues in the *k*‐th template that are also present in the target protein according to the BLASTp alignment. Note that our method do not rely on binding site or catalytic site information of the target protein, as such site information is not generally available. Meanwhile, *bitscore_k_* (*q*), *ID_k_* (*q*), *K*(*q*), *K_site_* (*q*), and *SiteAligned_k_* (*q*) are for the subset of templates with EC number *q*. The weight between global and local similarity, *w_seq_*, is defined as:

$$\;w_{seq}=1-\prod_{k=1}^{K}\left(1-ID_{k}\right)$$

In the structure template search step, the input protein structure is searched by Foldseek through all training enzyme protein structures from AFDB using the following search parameters: “easy‐search –format‐output query, qlen, target, tlen, evalue, bits, alnlen, nident, qaln, qstart, taln, tstart, qtmscore, ttmscore –tmalign‐fast 1 ‐e 0.1 ‐s 7.5”. For EC number *q*, its structure template‐based prediction score is calculated as:

$$\begin{array}{ccc} & & Cscore_{str}\;\left(q\right)\\ & & \;=w_{str}\;\cdot \frac{\sum_{k=1}^{K\left(q\right)}ID_{k}\left(q\right)\cdot bitscore_{k}\left(q\right)\cdot TM_{k}\left(q\right)}{\sum_{k=1}^{K}ID_{k}\cdot bitscore_{k}\cdot TM_{k}}+\left(1-w_{str}\right)\\ & & \;\cdot \;\frac{\sum_{k=1}^{K_{site}\left(q\right)}ID_{k}\left(q\right)\cdot bitscore_{k}\left(q\right)\cdot SiteAligned_{k}\left(q\right)\cdot TM_{k}\left(q\right)}{\sum_{k=1}^{K_{site}}ID_{k}\cdot bitscore_{k}\cdot SiteAligned_{k}\cdot TM_{k}} \end{array}$$

Here, *TM_k_* is the TM‐score between the target protein and the k‐th template protein. *w_str_* uses the same formula as *w_seq_*, except that the sequence identities of the Foldseek‐detected structure templates rather than those of the MMseqs2‐detected sequence homologs are used for its calculation. If the target and template proteins have different lengths, there will be two TM‐score values corresponding to the two sequence lengths, in which case the minimum between the two TM‐score values is used. The only difference between *Cscore_seq_* (*q*) and *Cscore_str_* (*q*) is that the latter includes structural similarity (TM‐score) from structure alignment performed using the TM‐align mode of Foldseek. The general formula for *Cscore_seq_* (*q*) and *Cscore_str_* (*q*) are verified by our previous study [21] to be effective for function prediction. The sequence and structure template‐based scores are then combined linearly:

$$\begin{array}{c} Cscore_{template}\;\left(q\right)=w_{seq}\;\cdot Cscore_{seq}\left(q\right)+\left(1-w_{seq}\right)\cdot Cscore_{str}\left(q\right) \end{array}$$

### 4.2 Deep Learning‐Based Component of EZpred

The deep learning‐based component of EZpred (blue component in Figure 1) is inspired by our previous pipelines InterLabelGO [55] and ATGO [35]. This component starts with feeding the target protein sequence into the ESM C protein language model to generate a sequence embedding matrix from its last three hidden layers. Each layer produces a 1152 × *L* matrix, where *L* is the length of the target protein. The ESM C model can only process up to 2048 tokens. Thus, if *L* > 2048, only the first 2048 residues of the protein sequence are considered. This truncation does not affect the test of EZpred, as only 8 out of 720 test enzymes have >2048 residues, and all these long enzymes have their active/binding site residues in the first 2048 residues. Mean pooling of the 1152 × *L* matrix is applied along the sequence to generate an embedding vector with 1152 elements. These three vectors from mean pooling of the last three layers of ESM C are concatenated into a single embedding vector with 1152 × 3  =  3456 elements.

Meanwhile, the target protein is also searched through the sequence database consisting of training enzymes using MMseqs2 to identify sequence homologs (See above Methods section for template‐based component of EZpred). Only the top four MMseqs2 hits are considered. Each of these four sequence homologs are passed through ESM C, and the embeddings from the last three layers of ESM C are mean pooled into an embedding vector with 3456 elements as above. These four embedding vectors, denoted as ${\overset{⇀}{E}}_{1}$ to ${\overset{⇀}{E}}_{4}$, are combined into a single embedding vector $\overset{⇀}{E}$ with 3456 elements by the following formula weighted averaging:

$$\;\overset{⇀}{E}=\frac{1}{\sum_{k=1}^{4}bitscore_{k}\cdot ID_{k}}\;\sum_{k=1}^{4}bitscore_{k}\cdot ID_{k}\cdot {\overset{⇀}{E}}_{k}$$

The combined embedding vector $\overset{⇀}{E}$ and the embedding vector from the target protein itself are concatenated into a single embedding vector with 3456 × 2  =  6912 elements. It is further processed by a feed forward neural network with the Gaussian Error Linear Unit (GELU) activation function [67] to transform the ESM C‐derived features into EC number probability. Based on predicted labels, we train two types of neural network models. The first type of model predicts 5586 EC numbers, including 7 EC numbers with 1 digit, 76 EC numbers with the first 2 digits, 267 EC numbers with the first 3 digits and 5201 EC numbers with all 4 digits. This is smaller than the total number of EC numbers (∼7000) as not all EC numbers correspond to at least one training enzyme with experimentally verified catalytic activity. The second type of model predicts all the above EC numbers as well as an additional label (0.‐.‐.‐) for non‐enzyme proteins. Since we use 5‐fold cross validation to optimize hyperparameters, we generate 5 models for each model type. The prediction scores for EC numbers and for non‐enzymes are derived from averaging the prediction result from the 5 models of the first type and second type, respectively. We use a dropout rate of 30% and a batch size of 512. Early stopping is applied if a model is trained with at least 20 epochs and the validation loss has not been improved for the last 10 epochs (Figure S9).

Similar to InterLabelGO, the neural network of EZpred is trained using a composite loss function consisting of the protein‐centric F1‐score, the EC number‐centric F1‐score, and the Zero‐bounded Log‐sum‐exp and Pairwise Rank‐based (ZLPR) loss [68]. Since the differences in the probability of different EC numbers are large, we use information content (IC, also known as information accretion) [69] to weigh different EC numbers, where the IC for EC number *q* is defined as:

$$IC\;\left(q\right)=\log_{2}\left(\frac{1+N\left(parent\left(q\right)\right)}{1+N\left(q\right)}\right)$$

Here, *N*(*q*) is the total number of training enzymes with EC number *q*; *parent*(*q*) is the parent of EC number *q*, i.e., the EC number with one less digit. For example, for EC number 2.7.4.3 “Adenylate kinase”, its parent EC number is 2.7.4.‐ “Phosphotransferases with a phosphate group as acceptor”. If *q* only has one digit, *N*(*parent*(*q*)) equals the number of all training enzymes. Using the IC, we define the IC‐weighted precision and recall for protein *t*:

$$\;wPrecision_{t}=\frac{\sum_{q=1}^{Q}y_{true}\left(t,q\right)\cdot y_{pred}\left(t,q\right)\cdot IC\left(q\right)}{\varepsilon +\sum_{q=1}^{Q}y_{pred}\left(t,q\right)\cdot IC\left(q\right)}$$

$$\;wRecall_{t}=\frac{\sum_{q=1}^{Q}y_{true}\left(t,q\right)\cdot y_{pred}\left(t,q\right)\cdot IC\left(q\right)}{\varepsilon +\sum_{q=1}^{Q}y_{true}\left(t,q\right)\cdot IC\left(q\right)}$$

Here, ε = 1E‐16 is a small value to avoid division by zero; *y_true_* (*t*,*q*) indicates whether protein *t* has EC number *q*; and *y_pred_* (*t*,*q*) is the predicted probability of EC number *q* for protein *t*. Q = 928 is total number of labels, i.e., EC numbers. Similarly, the precision and recall can also be defined for EC number *q*:

$$w\;Precision_{q}=\frac{\sum_{t=1}^{T}y_{true}\left(t,q\right)\cdot y_{pred}\left(t,q\right)\cdot IC\left(q\right)}{\varepsilon +\sum_{q=1}^{Q}y_{pred}\left(t,q\right)\cdot IC\left(q\right)}$$

$$w\;Recall_{q}=\frac{\sum_{t=1}^{T}y_{true}\left(t,q\right)\cdot y_{pred}\left(t,q\right)\cdot IC\left(q\right)}{\varepsilon +\sum_{q=1}^{Q}y_{true}\left(t,q\right)\cdot IC\left(q\right)}$$

Here, *T* is the total number of training proteins. The protein‐centric F1‐score loss can be defined as:

$$\;F1_{protein}=1-\frac{2}{T/\sum_{t=1}^{T}wPrecision_{t}+T/\sum_{t=1}^{T}wRecall_{t}}$$

Similarly, the EC‐centric F1‐score loss can be defined as:

$$\;F1_{EC}=1-\frac{2}{Q/\sum_{q=1}^{Q}wPrecision_{q}+Q/\sum_{q=1}^{Q}wRecall_{q}}$$

The third term for the loss function is the ZLPR loss [68], which was found in our previous study [55] to be a more effective replacement compared to the traditional binary cross entropy loss. The ZLPR loss is defined as:

$$\begin{array}{ccc} L_{zlpr} & = & log\;\left[1+\sum_{\left(t,q\right)\in \Omega_{pos}}\exp\left(-y_{pred}\left(t,q\right)\right)\right]\\ & & +\;\log\left[1+\sum_{\left(t,q\right)\in \Omega_{neg}}\exp\left(y_{pred}\left(t,q\right)\right)\right] \end{array}$$

Here, Ω_*pos*_ and Ω_*neg*_ are the sets of positive and negative protein‐EC number associations. The final composite loss function used to train EZpred is:

$$\;Loss_{final}=L_{zlpr}\;\cdot L_{F1EC}\cdot \sqrt{L_{F1protein}}$$

To ensure the true‐path rule, i.e., that the predicted probability of EC number *q*, *Cscore*(*q*), is no greater than the predicted probability of parent EC number *Cscore*(*parent*(*q*)), and no less than the maximum predicted probability of any of its child EC number *Cscore*(*child*(*q*)), an iterative post‐processing procedure is applied. In each iteration, the following steps are applied. If *Cscore*(*parent*(*q*)) ≥ *Cscore*(*q*) ≤ *Cscore*(*child*(*q*)), *Cscore*(*q*) is increased to max(*Cscore*(*parent*(*q*)), *Cscore*(*child*(*q*))). If *Cscore*(*parent*(*q*)) ≤ *Cscore*(*q*) ≥ *Cscore*(*child*(*q*)), *Cscore*(*q*) is decreased to min(*Cscore*(*parent*(*q*)), *Cscore*(*child*(*q*))). If *q* has all 4 digits and no child EC number and *Cscore*(*parent*(*q*)) < *Cscore*(*q*), *Cscore*(*q*) is decreased to the value of *Cscore*(*parent*(*q*)). If *q* has only 1 digit and no parent EC number, and *Cscore*(*q*) < *Cscore*(*child*(*q*)), *Cscore*(*q*) is decreased to the value of *Cscore*(*child*(*q*)). If *Cscore*(*parent*(*q*)) < *Cscore*(*q*) < *Cscore*(*child*(*q*)), *Cscore*(*q*) is not updated in the current iteration. The above iteration is performed up to 4 times, until the predicted probability of any EC number is no greater than that of its parent.

The template‐based score *Cscore_template_* (*q*) is then combined with the deep learning‐based prediction ***Cscore***_***DL***_(***q***) to derive the final EZpred prediction score for EC number *q*:

$$\begin{array}{ccc} & & Cscore_{EZpred}\;\left(q\right)\\ & & \;=w_{DL}\;\cdot Cscore_{DL}\left(q\right)+\left(1-w_{DL}\right)\cdot Cscore_{template}\left(q\right) \end{array}$$

The weight to balance the two components equals to *w_DL_* =  0.2 + 0.1 · (1 − *w_seq_*). Here, the values 0.2 and 0.1 are hyperparameters optimized by a grid search through cross‐validation.

## Author Contributions

C.Z. and L.F. conceived the project and designed the experiments. Q.L. and C.Z. developed the methods and performed the experiments. C.Z. curated the data. Q.L., C.Z. and L.F. analyzed the data and wrote the paper.

## Conflicts of Interest

The authors declare no conflicts of interest.

## Supporting information




**Supporting File**: advs78703‐sup‐0001‐SuppMat.docx.

## Data Availability

Data sharing not applicable to this article as no datasets were generated or analysed during the current study.
