# Supplementary material for: EZpred: Improving Deep Learning‐Based Enzyme Function Prediction Using Unlabeled Sequence Homologs
Source: Adv Sci (Weinh). 2026 Nov 10:e78703. Online ahead of print. doi: 10.1002/advs.78703 (PMC13750008; doi:10.1002/advs.78703)
Supplement: Supplementary file 1 [file ADVS-9999-e78703-s001.docx]

**Supporting Information for**

EZpred: improving deep learning-based enzyme function prediction using unlabeled sequence homologs

Quancheng Liu, Chengxin Zhang, Lydia Freddolino

C.Z. and L.F.

Email: [cx.zhang2@siat.ac.cn](mailto:cx.zhang2@siat.ac.cn) and [lydsf@umich.edu](mailto:lydsf@umich.edu)

**This PDF file includes:**

Supporting text

Figures S1 to S9

Tables S1 to S5

Fig. S1. Distribution of major enzyme classes (classified by the first digit of EC number), taxonomy division, and sequence length of enzymes in the test set and the training set of EZpred. In the taxonomy division, MAM stands for mammals excluding primates and rodents; VRT stands for non-mammalian vertebrates; and VRL stands for viruses excluding phages.

Fig. S2. Precison-recall curves of different methods to predict the first 3 and all 4 digits of EC numbers. Each curve has a marker to indicate the precision-recall combination that leads to the maximum F1-score. The values inside the legend are the Area Under Precision Recall Curve (AUPRC) values. DeepECtransformer and DeepEC do not provide a prediction score associated with the predicted EC numbers. Their precision-recall curves are thus shown as a single dot.

Fig. S3. F1-scores for predicting the 4-digit EC number on the test set. A. Evaluation using the EC number definition of EZpred. B. Evaluation using the EC number definition of CLEAN. The error bars indicate the standard error of mean (SEM) of per-target F1-score.

Fig. S4. F1-scores for predicting 4-digit EC numbers with different frequencies among training set enzymes. The error bars indicate the standard error of mean (SEM) of per-protein F1-score values.

Fig. S5. F1-scores of deep learning component of EZpred when using different number of sequence homologs and different protein language models (ESM C versus ESM2) for predicting the four-digit EC number on the test set. Error bars indicate the standard error of mean (SEM) for per-protein F1-scores.

Fig. S6. Distribution of 64 test enzymes for which F1-scores for 4-digit EC number prediction are different between deep learning and template-based components of EZpred.

Fig. S7 Comparison of prediction score of the deep learning component of EZpred for the true positive 4-digit EC numbers for wild type (WT) enzymes and corresponding mutants whose catalytic site residues are mutated to glycine. The comparison is performed on the subset of 177 test enzymes with known catalytic sites.

Fig. S8 Comparison of prediction score for human aldehyde dehydrogenase ALDH2 (UniProt accession: P05091) for EC 1.2.1.3 “aldehyde dehydrogenase (NAD(+))” by EZpred, CLEAN and GraphEC. Residues for benign mutations (I41L, I41V, P92A, P92T, R94H, T244M, V304M) are shown as green sticks. The residue for pathogenic mutation (E504K) is shown in red stick. The substrate NAD(+) is shown as spheres.

Fig. S9. Training/validation loss versus the number of epochs in EZpred training.

Table S1. F1-scores for predicting 4-digit EC numbers for the full test set, and for the 7 major classes of enzymes. The highest value (including ties) in each column is shown in bold text.

| Method | Cov-  erage | F1-score for predicting 4-digit EC numbers | | | | | | | | p-value |
| --- | --- | --- | --- | --- | --- | --- | --- | --- | --- | --- |
|  |  | EC 1.x.x.x oxido- reductases | EC 2.x.x.x transferases | EC 3.x.x.x hydrolases | EC 4.x.x.x lyases | EC 5.x.x.x isomerases | EC 6.x.x.x ligases | EC 7.x.x.x translocases | All |  |
| EZpred | 0.999 | **0.591** | **0.858** | **0.892** | **0.747** | **0.652** | 0.554 | **0.590** | **0.786** | * |
| CLEAN | **1** | 0.514 | 0.651 | 0.755 | 0.517 | 0.455 | **0.606** | 0.500 | 0.621 | 3.51E-20 |
| GraphEC | **1** | 0.438 | 0.700 | 0.694 | 0.470 | 0.461 | 0.447 | 0.210 | 0.600 | 2.89E-31 |
| ProteInfer | 0.493 | 0.334 | 0.713 | 0.479 | 0.702 | 0.138 | 0.519 | 0 | 0.559 | 1.35E-46 |
| ECPICK | 0.524 | 0.454 | 0.714 | 0.435 | 0.426 | 0.238 | 0.531 | 0.400 | 0.568 | 2.58E-45 |
| CLEAN-contact | **1** | 0.447 | 0.662 | 0.678 | 0.362 | 0.274 | 0.557 | 0.500 | 0.591 | 5.29E-17 |
| DeepECtransformer | 0.700 | 0.377 | 0.672 | 0.560 | 0.491 | 0.157 | 0.372 | 0.105 | 0.549 | 1.06E-37 |
| ECPred | 0.406 | 0.087 | 0.644 | 0.500 | 0.286 | 0.077 | 0.298 | 0 | 0.456 | 3.73E-63 |
| DeepEC | 0.372 | 0.285 | 0.590 | 0.277 | 0.279 | 0.138 | 0.494 | 0 | 0.425 | 3.15E-67 |

* p-value is calculated using single-tailed Wilcoxon rank sum test to determine whether EZpred has a significantly higher F1-score for predicting 4-digit EC numbers for the full test set.

Table S2. Total inference time of EZpred for the full test set consisting of 720 enzymes.

| EZpred components * | Running time (seconds),  averaged over 10 parallel runs |
| --- | --- |
| EZpred (template) using MMseqs2 templates only | 4.0 |
| EZpred (template) using MMseqs2 and Foldseek templates | 57.6 |
| EZpred (DL) without ESM C features from sequence homologs | 16.8 |
| EZpred (DL) with ESM C features from MMseqs2 homologs | 38.6 |
| EZpred, full pipeline | 96.0 |

* EZpred was run on 24 CPU cores from Intel® Core™ Ultra 9 Processor 285K. We ran the ESM C protein language model on a Nvidia RTX PRO 6000 Blackwell GPU to extract sequence features.

Table S3. F1-scores for predicting EC numbers for “hard” test targets. The best performance in each column (within each test set) is shown in bold.

| Test set | Methods | F1-score for predicting EC numbers | | | |
| --- | --- | --- | --- | --- | --- |
|  |  | First digit | First 2 digits | First 3 digits | All 4 digits |
| Hard enzymes with sequence identity≤0.3 to any training  protein | EZpred | 0.850 | **0.809** | 0.778 | 0.627 |
|  | EZpred (DL) | 0.824 | 0.797 | **0.781** | 0.600 |
|  | EZpred (template) | **0.856** | 0.803 | 0.777 | **0.636** |
|  | CLEAN | 0.826 | 0.730 | 0.666 | 0.468 |
|  | GraphEC | 0.640 | 0.588 | 0.555 | 0.456 |
|  | ProteInfer | 0.638 | 0.568 | 0.470 | 0.324 |
|  | ECPICK | 0.337 | 0.324 | 0.302 | 0.218 |
|  | CLEAN-Contact | 0.765 | 0.708 | 0.632 | 0.426 |
|  | DeepECtransformer | 0.575 | 0.532 | 0.498 | 0.349 |
|  | ECPred | 0.708 | 0.562 | 0.480 | 0.300 |
|  | DeepEC | 0.171 | 0.154 | 0.147 | 0.125 |
| Very hard enzymes with sequence identity≤0.3 and TM-score≤0.6 to any training  protein | EZpred | 0.769 | **0.795** | 0.756 | **0.689** |
|  | EZpred (DL) | 0.780 | 0.725 | **0.761** | 0.639 |
|  | EZpred (template) | 0.684 | 0.744 | 0.720 | 0.658 |
|  | CLEAN | **0.796** | 0.591 | 0.591 | 0.537 |
|  | GraphEC | 0.650 | 0.546 | 0.546 | 0.546 |
|  | ProteInfer | 0.414 | 0.414 | 0.333 | 0.276 |
|  | ECPICK | 0.345 | 0.345 | 0.345 | 0.276 |
|  | CLEAN-Contact | 0.642 | 0.583 | 0.542 | 0.406 |
|  | DeepECtransformer | 0.611 | 0.556 | 0.556 | 0.500 |
|  | ECPred | 0.537 | 0.214 | 0.214 | 0.214 |
|  | DeepEC | 0.214 | 0.214 | 0.214 | 0.214 |

Table S4. Performance of enzyme/non-enzyme classification. The highest value in each column (for each test set) is shown in bold.

| Negative set | Methods | Predicting enzymes | |  | Predicting non-enzymes | |
| --- | --- | --- | --- | --- | --- | --- |
|  |  | Recall | F1-score |  | Recall | F1-score |
| 625 proteins with MF GO term for non-enzyme activity in UniProt 2024_02 but without such terms in UniProt 2023_02 | EZpred | 0.927 | **0.942** |  | 0.950 | **0.932** |
|  | EZpred (DL) | 0.908 | 0.936 |  | **0.962** | 0.928 |
|  | EZpred (binary) ^†^ | 0.887 | 0.923 |  | 0.957 | 0.914 |
|  | EZpred (template) | **0.964** | 0.927 |  | 0.861 | 0.904 |
|  | DeepECtransformer | 0.726 | 0.753 |  | 0.757 | 0.725 |
|  | ECPred | 0.869 | 0.909 |  | 0.949 | 0.901 |
| 377 proteins with annotation scores of 4 and 5 and no EC numbers in UniProt 2024_02. All proteins are absent in UniProt 2023_02 | EZpred | 0.927 | 0.913 |  | 0.793 | 0.818 |
|  | EZpred (DL) | 0.908 | 0.911 |  | 0.830 | 0.825 |
|  | EZpred (binary) ^†^ | 0.887 | 0.903 |  | 0.846 | 0.817 |
|  | EZpred (template) | **0.964** | **0.971** |  | **0.955** | **0.942** |
|  | DeepECtransformer | 0.726 | 0.790 |  | 0.777 | 0.669 |
|  | ECPred | 0.869 | 0.899 |  | 0.873 | 0.817 |

^†^ EZpred (binary) refers to a deep learning model of EZpred trained as a simple binary classifier to predict whether a protein is an enzyme or a non-enzyme.

**Table S5.** Mean and standard deviation of per-target F1-scores of the deep learning models of EZpred for predicting the full 4-digit EC number on 720 test enzymes.

| EZpred (DL) versions | F1-score ^§^ | Mean of per-target F1-scores | Standard deviation of per-target F1-scores |
| --- | --- | --- | --- |
| Model 1 ^†^ | 0.750 | 0.671 | 0.444 |
| Model 2 | 0.750 | 0.658 | 0.462 |
| Model 3 | 0.760 | 0.679 | 0.448 |
| Model 4 | 0.755 | 0.660 | 0.458 |
| Model 5 | 0.735 | 0.653 | 0.452 |
| Final deep learning model | **0.782** | **0.698** | **0.443** |

† Model 1 to 5 refers to the five models obtained during 5-fold cross-validation of EZpred (DL). The final deep learning model was obtained by averaging the prediction results of Model 1 to 5.

§ The overall F1-score is calculated as $F1=2/\left( T/\sum_{t=1}^{T} {Precision}_{t}+T/\sum_{t=1}^{T} {Recall}_{t} \right)$, where ${Precision}_{t}$ and ${Recall}_{t}$ are the precision and recall of the *t*-th test protein among all *T* test proteins. The per-target F1-score for the *t*-th test protein is ${F1}_{t}=2/\left( 1/{{Precision}_{t}}+1/{{Recall}_{t}} \right)$. Through the Cauchy-Schwarz inequality, it can be easily proved that the mean of per-target F1-scores is less than or equal to the overall F1-score, i.e., $1/T\sum_{t=1}^{T} {F1}_{t}\leq F1$, and that equality holds if and only if $\forall t,{Precision}_{t}={Recall}_{t}$.
